# Quality of life 10 years after cardiac surgery in adults: a long-term follow-up study

**DOI:** 10.1186/s12955-020-01642-3

**Published:** 2020-12-10

**Authors:** Josip Vincelj, Lela Bitar

**Affiliations:** 1 Faculty of Medicine Osijek, University Josip Juraj Strossmayer Osijek, Josipa Huttlera 4, 31000 Osijek, Croatia; 2 Polyclinic for Cardiology „BOGDAN“, Bužanova 4, 10000 Zagreb, Croatia; 3 Department for Pulmonary Diseases, University Hospital Center Zagreb, Jordanovac 104, 10000 Zagreb, Croatia

**Keywords:** Quality of life, Coronary artery bypass graft, Follow up

## Abstract

We have read the article titled „Quality of life 10 years after cardiac surgery in adults: a long-term follow-up study “ by Perrotti A. et al. published in your distinguished journal with great interest. Unfortunately, we found few errors in this article.

Dear Editor,

I am contacting you in the name of the group of authors who published “Health-related quality of life five years after coronary artery bypass graft surgery” in the International Journal of Cardiology [1]. We read the article "Quality of life 10 years after cardiac surgery in adults: a longterm follow-up study“ by Perrotti A. et al., published in Health and Quality of Life Outcomes [2]. In Perrotti et al.'s discussion, the authors wrote that reference number 28 pertained to a population of 140 patients who underwent coronary artery bypass graft (CABG) in Poland. In the following sentence, Perrotti et al. also wrote that the patients in reference number 28 were from Poland. However, reference number 28 is actually our study [1] and we would like to correct the record and indicate that the 140 patients were Croatian and recruited from Dubrava University Hospital, Zagreb.

## Abbreviation

CABG: Coronary artery bypass graft
